# CXCL10^high^ Microglia in Cerebral Malaria: Toward Translational Validation

**DOI:** 10.1002/cns.70742

**Published:** 2026-01-10

**Authors:** Nathkapach K. Rattanapitoon, Chadaporn N. Gordon, Natthawut Charoenphon, Schawanya K. Rattanapitoon

**Affiliations:** ^1^ FMC Medical Center Nakhon Ratchasima Thailand; ^2^ Faculty of Allied Health Sciences Burapha University Chonburi Thailand; ^3^ Faculty of Medical Science Naresuan University Pitsanulok Thailand

## Abstract

Wang et al. identify CXCL10^high^ TNFα^high^ Ki67^+^ microglia as drivers of CD8^+^ T cell recruitment during experimental cerebral malaria (ECM). We propose a conservative reframing: rather than asserting a fully validated new taxonomy, CXCL10^high^ microglia should be considered a candidate “neuroimmune endotype” whose translational relevance requires clearly prespecified and testable validation in human disease. Priority validation steps include single‐nucleus and spatial transcriptomics of human postmortem tissue, paired plasma/CSF biomarker correlation, and mechanistic assays of microglial antigen presentation. We caution against strong therapeutic inferences from ECM alone; translational work should first establish reproducible human microglial signatures and clinical correlations before precision‐targeted interventions are proposed.

Dear Editor,

The elegant study by Wang et al. [1] identifies a distinct microglial population—CXCL10^high^ TNFα^high^ Ki67^+^—that recruits and sustains CD8^+^ T cell activity during experimental cerebral malaria (ECM). We welcome this novel observation and suggest a cautious and pragmatic pathway to explore whether this murine signature might represent a clinically meaningful neuroimmune endotype in human cerebral malaria (CM). Below, we (i) outline practical criteria for defining such an endotype, (ii) propose testable translational steps that directly build on Wang et al.'s findings, and (iii) briefly acknowledge therapeutic considerations as longer‐term possibilities contingent upon human validation.

## 1 Defining a Neuroimmune Endotype—Operational Criteria (Testable)

To move beyond conceptual language, we propose four minimal, testable criteria that a CXCL10^high^ microglial endotype should ideally meet in human CM:

- Reproducible CNS signature: a conserved transcriptional program (e.g., CXCL10^high^/TNFα^high^ proliferation markers) detectable by snRNA‐seq or spatial transcriptomics in multiple human CM brain samples [2];
- Peripheral biomarker linkage: a reproducible association between this CNS signature and a measurable plasma/CSF biomarker pattern (e.g., chemokine profile) in independent cohorts [3];
- Clinical correlation: statistical association between the CNS signature (or its peripheral surrogate) and relevant outcomes (acute severity, death, or validated long‐term neurocognitive impairment) after appropriate adjustment for known confounders [3];
- Mechanistic plausibility: corroborative mechanistic data, such as evidence for antigen presentation or chemokine‐mediated recruitment, acquired using targeted assays [4].

Collectively, fulfillment of these criteria would support the classification of CXCL10^high^ microglia as a bona fide neuroimmune endotype rather than a preliminary preclinical observation.

## 2 Concrete Translational Steps That Follow From Wang Et al.

We suggest three near‐term, testable approaches that build on Wang et al.'s data and address concerns regarding cross‐species extrapolation:

- Human postmortem snRNA‐seq and spatial transcriptomics: application of standardized analytical pipelines to multiple human CM brain samples (ideally from different geographic settings) to identify a microglial transcriptional module analogous to the murine CXCL10^high^ program [2].
- Paired plasma/CSF profiling in acute CM survivors: evaluation of whether peripheral chemokine signatures correlate with CNS microglial states (or with postmortem CNS signatures when available), using proteomic approaches and targeted chemokine panels, alongside assessment of associations with neurological outcomes [3].
- Mechanistic assays in ECM refined to test antigen presentation directly: for example, the use of MHC‐I tetramers, in vivo antigen tracing, and conditional microglial MHC‐I manipulation to clarify whether microglia present parasite antigens to CD8^+^ T cells or primarily function as chemokine sources [4].

These steps are intended to generate human‐centered, testable evidence while keeping speculation to a minimum.

## 3 Cross‐Species Caveats and Measured Interpretation

We recognize that ECM and human CM differ in parasite species, sequestration patterns, and immune thresholds; consequently, translational interpretations should remain appropriately cautious and framed as hypotheses requiring validation rather than as established equivalence. For instance, although peripheral CXCL10 elevations have been associated with poorer outcomes in some clinical series, the existing evidence remains heterogeneous and does not yet demonstrate that plasma CXCL10 reliably reflects a CNS microglial state predictive of long‐term deficits [3]. Accordingly, such associations should be interpreted in a confirmatory rather than deterministic manner.

## 4 Therapeutic Implications—Restrained and Conditional

Therapeutic concepts inspired by ECM (e.g., CXCL10/CXCR3 modulation, nanoparticle delivery, or metabolic reprogramming) are biologically plausible; however, they remain speculative in the absence of validated human evidence for the proposed endotype. We therefore suggest a phased approach: generation of reproducible human data confirming the endotype and target engagement, followed by preclinical safety and efficacy studies incorporating human tissue‐derived readouts, and subsequently the consideration of carefully designed, biomarker‐stratified early‐phase trials [5, 6].

## 5 How This Letter Extends Wang Et al.'s Work

Rather than seeking to replace the conclusions of Wang et al., our aim is to translate their experimental insights into an operational research agenda that may be examined in human CM. Specifically, we highlight experimental strategies (snRNA‐seq on human tissue, paired plasma/CSF correlation, and antigen‐presentation assays) that could directly test key mechanistic hypotheses and facilitate a considered bridge from murine observations to human pathology [1, 2, 4].

Wang et al. provide important mechanistic insights into ECM microglial biology; viewing CXCL10^high^ microglia as a candidate neuroimmune endotype, guided by explicit operational criteria and a focused validation plan, may help advance rigorous translational investigation while avoiding premature therapeutic claims. We encourage collaborative, multicentre efforts integrating high‐resolution human CNS profiling with prospective biomarker and outcome studies to determine whether neuroimmune endotypes can meaningfully stratify CM patients and inform future precision interventions.

## Funding

The authors have nothing to report.

## Ethics Statement

The authors have nothing to report.

## Conflicts of Interest

The authors declare no conflicts of interest.

## Data Availability

Data sharing not applicable to this article as no datasets were generated or analyzed during the current study.

## References

[1] Y. Wang , J. Liang , C. Yang , et al., “CXCL10^high^ TNFα^high^ Ki67^+^ Microglia Recruit and Activate CD8^+^ T Cells in the Brainstem During Experimental Cerebral Malaria,” CNS Neuroscience & Therapeutics 31 (2025): e70425, 10.1111/cns.70425. 40457525 PMC12129709

[2] J. Chen , Y. Bai , X. He , et al., “The Spatiotemporal Transcriptional Profiling of Murine Brain During Experimental Cerebral Malaria,” Nature Communications 16, no. 1 (2025): 1540, 10.1038/s41467-024-52223-7. PMC11814382 39934099

[3] B. J. Ouma , M. A. Idro , A. L. Conroy , et al., “Elevated Angiopoietin‐2 Is Associated With Worse Neurocognitive Outcomes in Children Surviving Severe Malaria,” Malaria Journal 20 (2021): 334, 10.1186/s12936-020-03545-6.34330288

[4] S. Aydin , J. Pareja , V. M. Schallenberg , et al., “Antigen Recognition Detains CD8^+^ T Cells at the Blood–Brain Barrier and Contributes to Its Breakdown,” Nature Communications 14 (2023): 3106, 10.1038/s41467-023-38703-2. PMC10229608 37253744

[5] J. Miu , A. J. Mitchell , M. Müller , et al., “Chemokine Gene Expression During Fatal Murine Cerebral Malaria and Protection due to CXCR3 Deficiency,” Journal of Immunology 180, no. 2 (2008): 1217–1230, 10.4049/jimmunol.180.2.1217. 18178862

[6] Q. Zhang , S. S. Wang , Z. Zhang , and S. F. Chu , “PKM2‐Mediated Metabolic Reprogramming of Microglia in Neuroinflammation,” Cell Death Discovery 11 (2025): 149, 10.1038/s41420-025-02453-5. 40189596 PMC11973174

